# Layered double hydroxide-based ROS-scavenging nanozyme hydrogels re-establish osteoimmune-angiogenic coupling for osteoporotic bone repair

**DOI:** 10.1186/s12951-026-04879-0

**Published:** 2026-08-01

**Authors:** Bangguo Wei, Hong Wang, Yi Gao, Huichao Wang, Jiale Gao, Lining Wang, Jiaying Wu, Xijin Wang, Guoxin Jing, Shilong Wang

**Affiliations:** 1 Research Center for Translational Medicine at East Hospital, School of Life Sciences and Technology, Tongji University, Shanghai, 200092 PR China; 2 Department of Neurology, Shanghai Tongji Hospital, School of Medicine, Tongji University, Shanghai, 200065 PR China

**Keywords:** Composite nanoparticles, Composite hydrogel, Immunomodulation, Microenvironment remodeling, Osteoporotic bone defect

## Abstract

**Background:**

Osteoporotic bone defects (OP-BDs) present a significant clinical challenge because of a pathological microenvironment characterized by severe oxidative stress, persistent inflammation, and disrupted metabolic coupling between osteoblasts and osteoclasts. To overcome these barriers, we developed a microenvironment-remodeling nanocomposite hydrogel for re-establishing osteoimmunological and angiogenic homeostasis.

**Results:**

Manganese dioxide (MnO_2_) nanozymes were first deposited onto layered double hydroxides (LDHs) via an in situ redox reaction to fabricate MnO_2_@LDHs nanocomposites, which were subsequently integrated into a gelatin methacryloyl (GelMA) network through interfacial hydrogen bonding to form the final hydrogel (MnO_2_@LDHs/GelMA). In vitro assays confirmed that the MnO_2_ coating exhibited catalase-like activity, decomposing pathological hydrogen peroxide (H_2_O_2_) into water and oxygen (O_2_), thereby alleviating oxidative stress and protecting the LDH framework from acid-induced degradation. The resulting ROS depletion modulated macrophage polarization toward a pro-regenerative phenotype, thereby attenuating the inflammatory microenvironment and promoting endothelial cell migration and microvascular tube formation via paracrine signaling. Furthermore, the preserved LDH nanoplatform synergized with the ROS-depleted microenvironment to regulate bone metabolism, enhancing the osteogenic differentiation and matrix mineralization of bone marrow mesenchymal stem cells (BMSCs) while suppressing RANKL-induced osteoclastogenesis in bone marrow-derived macrophages (BMDMs). In vivo evaluations in an ovariectomized (OVX) rat critical-sized femoral defect model demonstrated that the MnO_2_@LDHs/GelMA hydrogel significantly attenuated inflammatory infiltration, re-established bone remodeling homeostasis, and accelerated vascularized bone regeneration.

**Conclusion:**

This microenvironment-remodeling strategy highlights the therapeutic potential of integrating nanozymes with two-dimensional (2D) nanoplatforms, offering a promising nanobiomaterial-based approach for osteoporotic bone repair.

**Graphical Abstract:**

**Figure:**
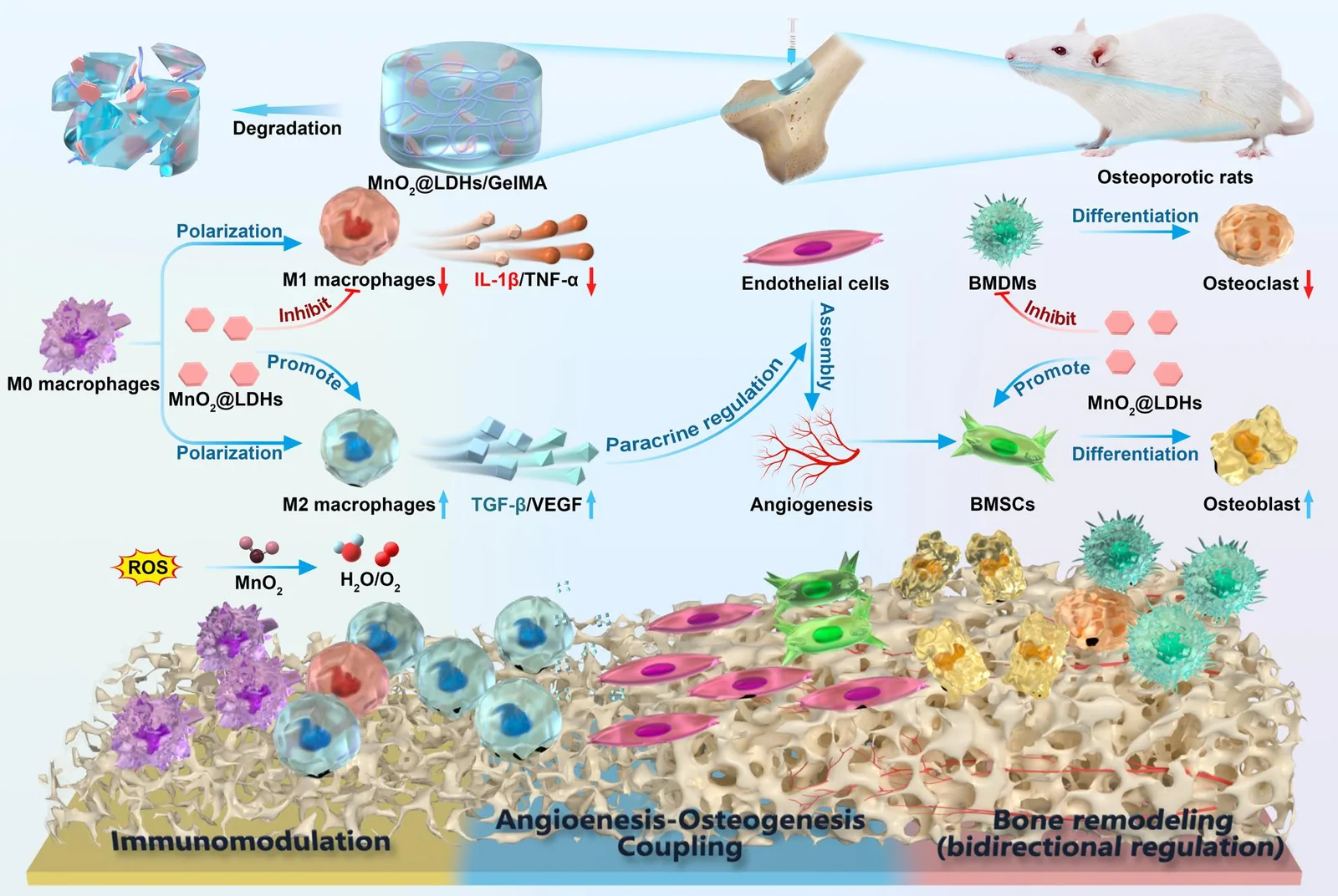

**Supplementary Information:**

The online version contains supplementary material available at https://doi.org/10.1186/s12951-026-04879-0.

## Introduction

Osteoporosis (OP) is a systemic metabolic bone disease characterized by a reduction in bone mineral density and the microarchitectural deterioration of bone tissue. These profound structural compromises substantially increase skeletal fragility, predisposing patients to fragility fractures, which often represent the first clinical event leading to symptom onset and diagnosis [1, 2]. In osteoporotic patients, fractures, trauma, or surgery disrupt bone integrity, leading to the formation of osteoporotic bone defects (OP-BDs) [3, 4]. Unlike routine injuries in healthy bones, the repair of OP-BDs goes beyond merely filling a physical void; it requires addressing a complex biological challenge driven by systemic metabolic abnormalities [5, 6].

Physiologically, the bone microenvironment is maintained through a dynamic equilibrium among key resident cells, particularly osteocytes, osteoblasts, and osteoclasts, which coordinate bone formation and bone resorption. Disruption of this tightly regulated balance leads to excessive bone resorption relative to bone formation, which is a central pathological feature of OP. However, in postmenopausal OP, estrogen deficiency acts as a major pathological driver that disrupts this delicate balance. This hormonal decline profoundly alters RANKL/OPG signaling, triggering increased osteoclast activity, systemic oxidative stress, and low-grade inflammatory changes [7, 8]. Specifically, the local microenvironment of OP-BDs is subjected to severe oxidative stress and persistent inflammation, often characterized by dysregulated immune responses mediated by senescent macrophages [9, 10]. The accumulation of reactive oxygen species (ROS) induces cytotoxicity, impairs vascular integrity, and disrupts the coupling between bone formation and bone resorption [11, 12]. These pathological cascades collectively result in suppressed osteogenic differentiation and compromised osseointegration. Consequently, traditional bone tissue engineering strategies—which primarily rely on passive physical support or the delivery of single osteogenic factors—often fail to achieve satisfactory bone regeneration [13]. Therefore, developing biomaterials capable of actively remodeling this pathological microenvironment has become a prerequisite for successful OP-BDs repair.

To address these multifaceted challenges, bioactive inorganic nanomaterials have garnered attention [14]. Among these, layered double hydroxides (LDHs) exhibit considerable therapeutic potential [15–17]. Our previous research demonstrated that nitrate-intercalated and strontium (Sr)-doped LDHs (Sr-LDHs) possess immunomodulatory and bone homeostasis-regulating capacities, showing efficacy in reversing the osteoporotic microenvironment [18–21]. However, Sr-LDHs exhibit inherent limitations when confronted with the OP-BDs milieu. Specifically, they lack intrinsic ROS-scavenging catalytic capability, and their structural integrity is vulnerable to the acidic conditions typically associated with inflammatory bone defects.

To overcome these limitations, given that hydrogen peroxide (H_2_O_2_) is the predominant ROS species generated during bone injury [22], surface modification with artificial nanozymes emerges as an effective strategy. Among various nanozymes with catalase-like ability, manganese dioxide (MnO_2_) was selected for this study due to its multidimensional advantages in bone tissue engineering. First, MnO_2_ nanomaterials possess catalase-like activity, catalyzing the decomposition of excess H_2_O_2_ into oxygen (O_2_) and water, thereby scavenging ROS, alleviating oxidative stress, and mitigating local hypoxia [23]. Second,manganese is an essential trace element for skeletal development, and the gradual degradation of the MnO_2_ coating releases bioactive manganese ions, which have been shown to promote osteogenic differentiation and stimulate angiogenesis. Finally, decorating the LDH surface with MnO_2_ (MnO_2_@LDHs) not only endows the platform with potent ROS-scavenging capabilities but also provides chemical protection in addition to serving as a physical barrier. In the localized acidic inflammatory microenvironment, the catalytic decomposition reaction of MnO_2_ inherently consumes local protons, thereby exerting an acid-buffering effect that fundamentally protects the underlying LDH layers from premature degradation. For localized in vivo delivery, these functional nanoparticles were incorporated into a biomimetic gelatin methacryloyl (GelMA) hydrogel network [24]. This integration establishes additional physical crosslinking sites, exerting a synergistic nano-reinforcement effect that enhances the mechanical stiffness and structural stability of the hydrogel.

**Scheme 1:**
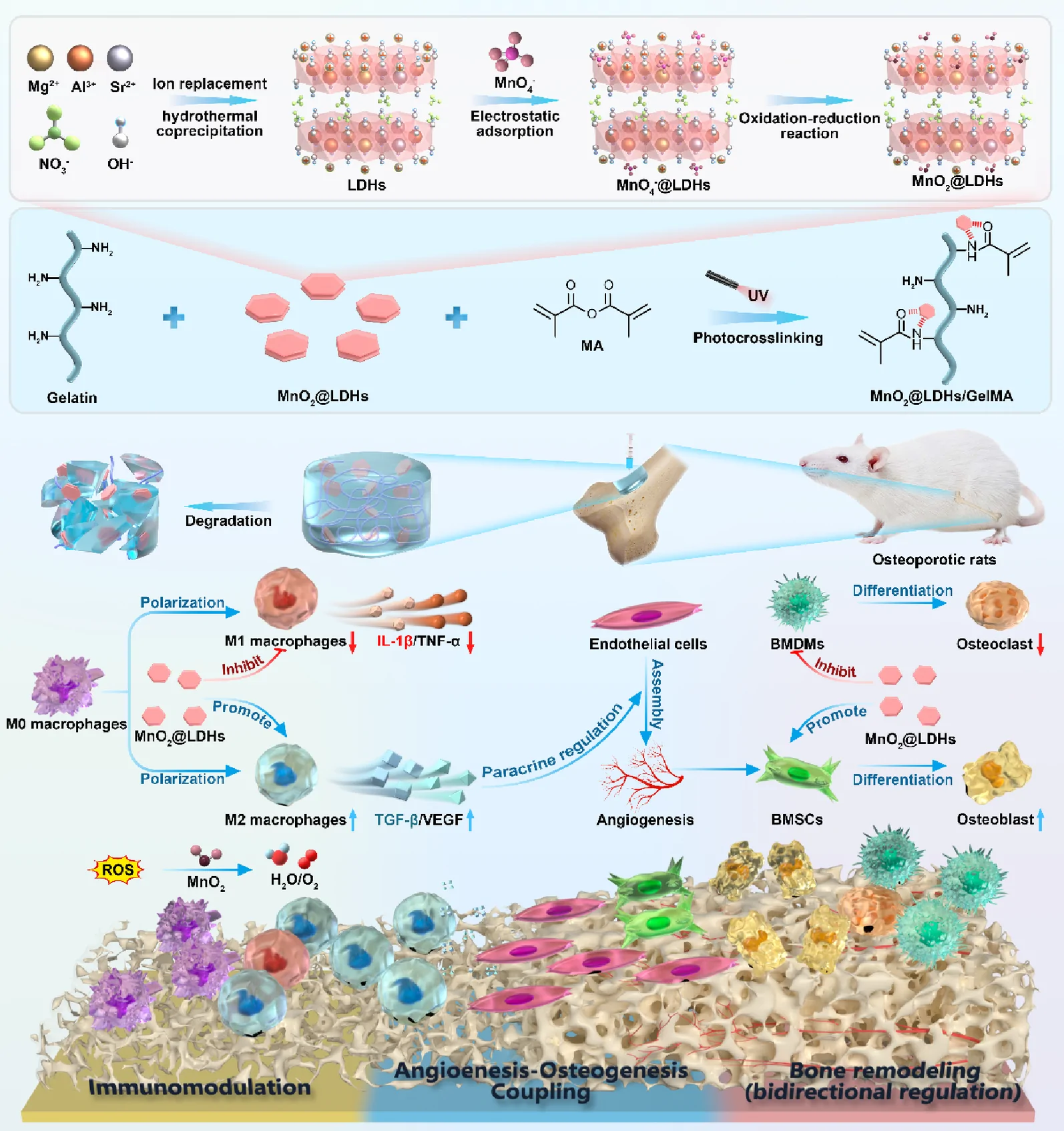
Schematic illustration of the microenvironment-remodeling MnO_2_@LDHs/GelMA hydrogel for osteoporotic bone defect repair

In this study, a microenvironment-remodeling nanocomposite hydrogel (MnO_2_@LDHs/GelMA) was developed to repair OP-BDs through the synergistic modulation of oxidative stress and the osteoimmune niche (Scheme 1). Systematic in vitro evaluations demonstrated that the hydrogel effectively scavenged ROS, thereby alleviating oxidative stress and protecting resident cells from oxidative damage. Moreover, the inherent bioactivity of the preserved LDH nanoplatform, together with the MnO_2_ nanozyme activity, exerted dual regulatory effects—promoting osteogenesis while concurrently inhibiting RANKL-induced osteoclastogenesis. Notably, the hydrogel attenuated the chronic inflammatory baseline by modulating macrophage polarization toward a pro-regenerative phenotype, thereby enhancing macrophage-mediated paracrine signaling to support angiogenesis. In vivo assessments in a rat OP-BDs model confirmed that the implanted hydrogel remodeled the local pathological osteoimmune microenvironment, accelerating vascularized bone regeneration and offering a promising translational biomaterial strategy for the clinical management of OP-BDs.

## Results

### Synthesis and characterization of MnO_2_@LDHs nanoparticles

**Fig. 1:**
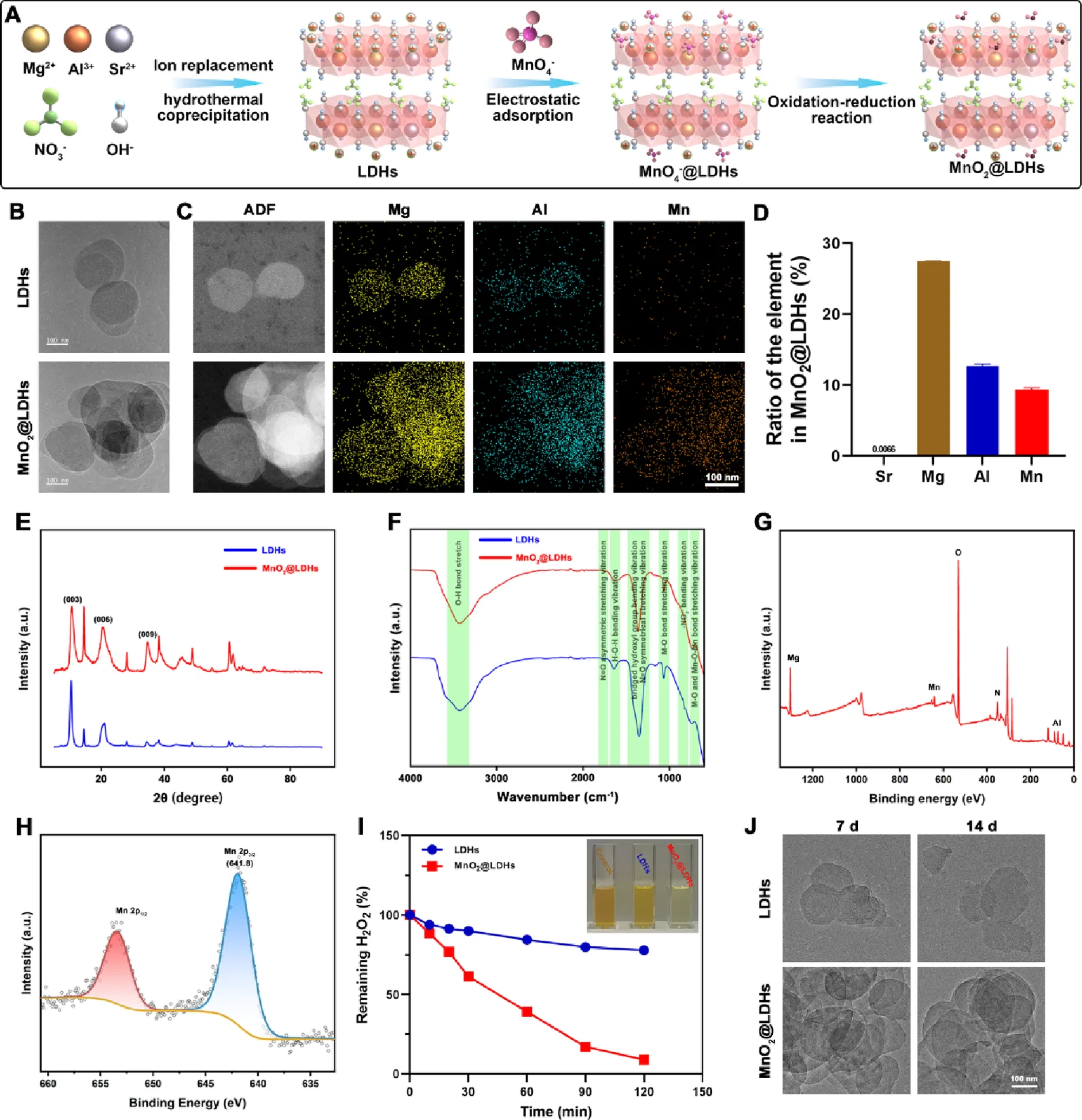
Characterization of LDHs and MnO_2_@LDHs nanocomposites. **(A)** Schematic diagram of MnO_2_@LDHs preparation. **(B)** TEM images of LDHs and MnO_2_@LDHs. **(C)** EDS elemental mapping of LDHs and MnO_2_@LDHs. **(D)** ICP-MS analysis of the elemental composition of MnO_2_@LDHs (*n* = 3). **(E)** XRD patterns and **(F)** FTIR spectra of LDHs and MnO_2_@LDHs. **(G)** XPS survey spectrum and **(H)** high-resolution Mn 2p XPS spectra of MnO_2_@LDHs. **(I)** Time-dependent H_2_O_2_ degradation profiles evaluated via the Ti(SO_4_)_2_ colorimetric assay. **(J)** Representative TEM images showing the in vivo morphological stability of LDHs and MnO_2_@LDHs at 7 and 14 days post-implantation

To develop a microenvironment-remodeling nanoplatform, we first synthesized MnO_2_@LDHs nanocomposites via an in situ redox deposition strategy (Fig. 1A). Transmission electron microscopy (TEM) revealed that the LDHs exhibited a characteristic two-dimensional (2D) nanosheet morphology (Fig. 1B). Following the in situ deposition, the MnO_2_@LDHs nanocomposites retained this lamellar structure. Energy-dispersive X-ray spectroscopy (EDS) mapping of the MnO_2_@LDHs demonstrated the uniform spatial distribution of Mg, Al, and Mn elements across the nanosheets (Fig. 1C). Furthermore, inductively coupled plasma mass spectrometry (ICP-MS) quantified the elemental composition of the MnO_2_@LDHs, yielding weight percentages of 27.4 wt% for Mg, 12.71 wt% for Al, and 9.38 wt% for Mn (Fig. 1D), corresponding to a Mg/Al molar ratio of approximately 2.4:1. These results confirm the successful incorporation and homogeneous distribution of Mn across the LDH nanosheets without compromising the structural integrity of the host framework.

To further elucidate the phase structure and chemical bonding of the synthesized nanocomposites, crystallographic and spectroscopic analyses were performed. X-ray diffraction (XRD) analysis showed that MnO_2_@LDHs retained the characteristic hydrotalcite-like basal reflections corresponding to the (003), (006), and (009) planes of LDHs (Fig. 1E). Lattice parameter calculations revealed that the (003) interlayer spacing (d_003_) decreased from 0.8548 nm in LDHs to 0.8308 nm in MnO_2_@LDHs. Meanwhile, no crystalline MnO_2_ diffraction peaks were detected. Fourier-transform infrared (FTIR) spectra revealed a Mn-O stretching vibration band at 737.25 cm^−1^ in the MnO_2_@LDHs (Fig. 1F). Additionally, the X-ray photoelectron spectroscopy (XPS) survey spectrum confirmed the elemental composition (Mg, Al, Mn, N, and O) of the composite (Fig. 1G), and the high-resolution Mn 2p spectrum exhibited a Mn 2p_3/2_ peak at 641.8 eV (Fig. 1H), consistent with the Mn (IV) oxidation state [25–27]. These data indicate the successful deposition of an amorphous MnO_2_ layer onto the LDH surface.

The pathological osteoporotic microenvironment is characterized by excessive ROS accumulation and localized acidic conditions, both of which pose significant challenges to biomaterial stability [28]. Therefore, we evaluated the catalytic ROS-scavenging capability and morphological stability of the nanoparticles. To quantify the ROS-scavenging capability, a titanium sulfate (Ti(SO_4_)_2_) colorimetric assay was employed to monitor H_2_O_2_ degradation (Fig. 1I). The LDHs group exhibited limited intrinsic catalase-like activity, with approximately 77.4% of H_2_O_2_ remaining after 120 min of incubation. In contrast, the MnO_2_@LDHs group demonstrated a rapid and time-dependent reduction in H_2_O_2_ concentration, with only approximately 8.5% remaining at the same time point. Consistently, the dissolved O_2_ generation assay (Fig. S1) revealed that whereas the LDHs group induced only a negligible increase in dissolved O_2_, the MnO_2_@LDHs group elicited a substantial and continuous increase. These findings demonstrate the efficient catalase-like activity of MnO_2_@LDHs for decomposing excess H_2_O_2_ into O_2_ and water. Moreover, morphological evaluation following in vivo implantation (Fig. 1J) showed that the LDHs lost their original lamellar structure and exhibited structural collapse by day 14 post-implantation, whereas MnO_2_@LDHs maintained their nanosheet morphological integrity. Collectively, these findings demonstrate that the MnO_2_@LDHs nanocomposite exhibits efficient catalase-like activity for ROS scavenging while maintaining enhanced structural stability under the pathological conditions.

### Fabrication and characterization of composite hydrogel

**Fig. 2:**
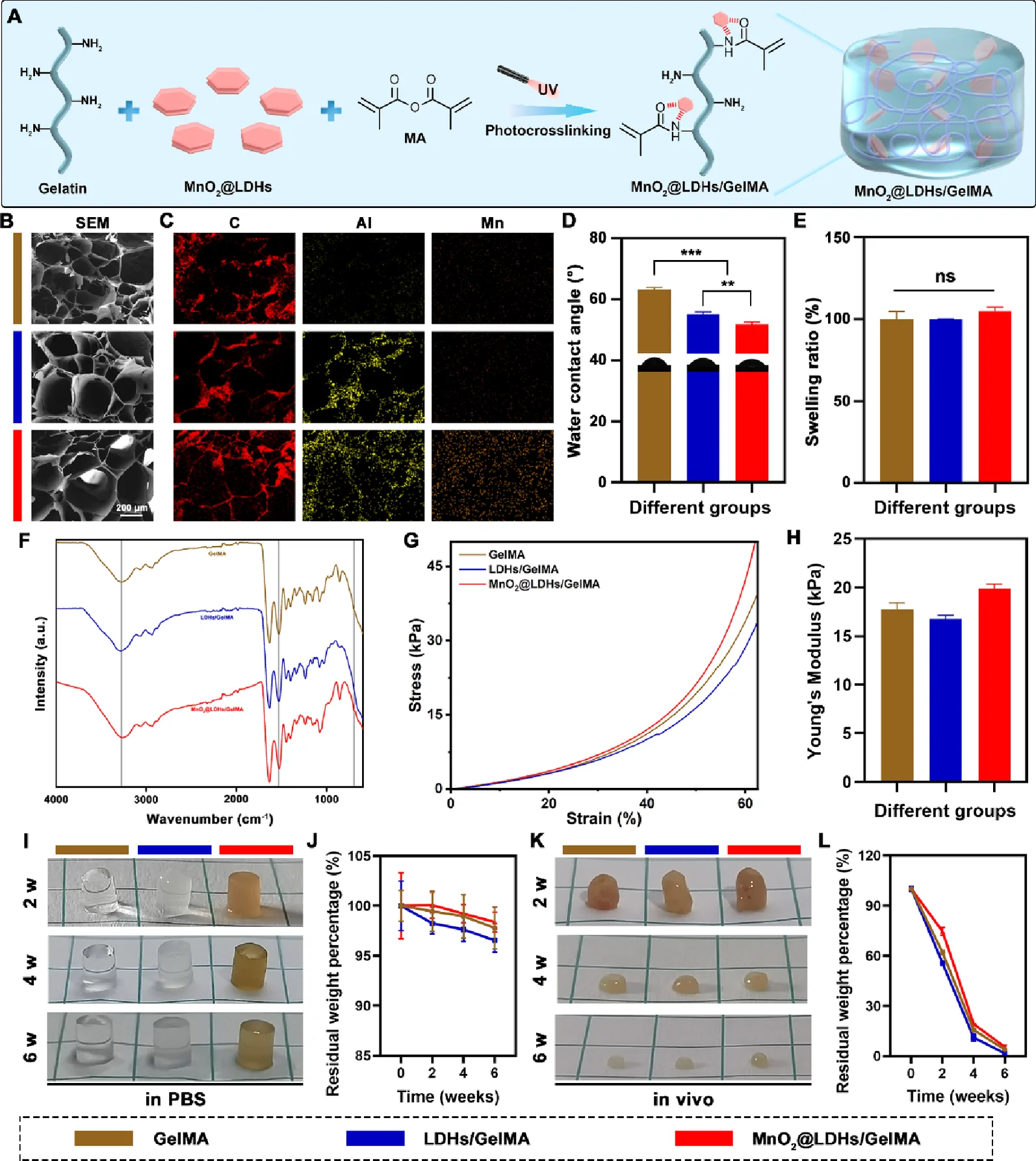
Characterization of the composite hydrogel. **(A)** Schematic illustration of MnO_2_@LDHs/GelMA preparation. **(B)** Cross-sectional SEM images and **(C)** EDS elemental mapping of different hydrogels. **(D)** Water contact angles and **(E)** equilibrium swelling ratios of different hydrogels (*n* = 3). **(F)** FTIR spectra of different hydrogels. **(G)** Compressive stress-strain curves and **(H)** Young’s modulus of different hydrogels (*n* = 3). **(I)** Macroscopic images and **(J)** residual mass of the hydrogels during in vitro degradation in PBS (*n* = 3). **(K)** Macroscopic images and **(L)** residual mass of the hydrogels during in vivo degradation in rat OP-BDs (*n* = 3)

The MnO_2_@LDHs/GelMA composite hydrogel was prepared by ultrasonically dispersing MnO_2_@LDHs into the GelMA precursor solution, followed by UV crosslinking (Fig. 2A). To balance biological cues and matrix structural integrity, various MnO_2_@LDHs loading concentrations were evaluated within the GelMA hydrogel. Based on evaluations of cytocompatibility, cytoskeletal spreading, and osteogenic gene expression (Fig. S2), the hydrogel containing 0.25% (w/v) MnO_2_@LDHs exhibited favorable cellular responses and was thus selected for all subsequent material characterization and biological evaluations.

Scanning electron microscopy (SEM) revealed that all hydrogels exhibited an interconnected, porous, honeycomb-like network structure without obvious structural collapse (Fig. 2B). EDS mapping of the MnO_2_@LDHs/GelMA hydrogel demonstrated the homogeneous co-localization of C, Al, and Mn elements throughout the scaffold matrix (Fig. 2C). Furthermore, the incorporation of the nanocomposites decreased the water contact angle from 63.22° in GelMA to 51.77° (Fig. 2D), while maintaining an equilibrium swelling ratio of approximately 100% across all groups (Fig. 2E). These results confirm the integration of the nanoparticles, which improved scaffold hydrophilicity without compromising the macroscopic porous architecture of the scaffold. To investigate the interfacial interactions underlying the mechanical performance of the composite hydrogel, FTIR spectra were analyzed (Fig. 2F). The characteristic O-H/N-H stretching vibration band exhibited a red shift from 3272.51 cm^-1^ in the GelMA hydrogel to 3262.52 cm^−1^ in the MnO_2_@LDHs/GelMA hydrogel, suggesting the formation of interfacial hydrogen bonds between the nanoparticles and the GelMA network. Consistent with the FTIR results, compression testing revealed that although the LDHs/GelMA hydrogel exhibited a slight reduction in both compressive strength and Young’s modulus compared with the GelMA hydrogel, the MnO_2_@LDHs/GelMA hydrogel exhibited the highest compressive strength and Young’s modulus among all groups (Fig. 2G and H). These findings suggest that the functionalized nanoparticles serve as additional physical crosslinking sites within the GelMA network, thereby enhancing the mechanical stiffness and structural stability of the hydrogel. To further evaluate the mechanical stability of the scaffolds during hydrolytic degradation, the compressive Young’s modulus was monitored over time (Fig. S3). As expected, all groups exhibited a gradual decline in compressive modulus due to the progressive hydrolytic degradation of the GelMA polymer network. Nevertheless, the MnO_2_@LDHs/GelMA hydrogel consistently maintained a significantly higher modulus than the GelMA and LDHs/GelMA hydrogels throughout the observation period. Even after 14 days of degradation, the nanocomposite hydrogel retained a compressive modulus of approximately 14.2 kPa.

Furthermore, the degradation profiles of the hydrogels were evaluated under both in vitro and in vivo conditions. Under in vitro physiological conditions (Fig. 2I and J), all hydrogel groups degraded slowly, maintaining more than 95% of their initial mass after 6 weeks of incubation. However, following implantation into the rat OP-BDs model, all hydrogels exhibited accelerated degradation kinetics (Fig. 2K and L). The residual mass of the implanted hydrogels decreased progressively over time, approaching complete degradation by week 6 post-implantation. This spatiotemporal degradation behavior suggests that the composite hydrogel provides physical support during the early stage of repair while gradually creating space for subsequent bone tissue ingrowth.

In parallel with the degradation study, the release kinetics of the bioactive ions (Mg^2+^ and Mn^2+^) from the composite hydrogel were monitored in PBS over 14 days (Fig. S4). As shown in the release profiles, Mg^2+^ exhibited a sustained and controlled release pattern, reaching a cumulative release of approximately 5.5% by day 14. This sustained release is primarily attributed to the gradual dissolution of the LDH framework, thereby providing continuous pro-osteogenic cues. In contrast, the cumulative release of Mn^2+^ was nearly undetectable, with only a negligible initial release (~0.75‱) on day 1, followed by no further release. This negligible Mn^2+^ release under neutral physiological conditions further demonstrates that the surface MnO_2_ layer remains highly stable and inert in a non-pathological environment, effectively preventing premature structural degradation and undesired Mn^2+^ leakage.

### Cytoprotection and intracellular ROS scavenging of the composite hydrogel

CCK-8 assays were performed on three key cell types involved in bone remodeling: bone marrow mesenchymal stem cells (BMSCs), bone marrow-derived macrophages (BMDMs), and human umbilical vein endothelial cells (HUVECs) (Fig. S5). The results showed no decrease in cell viability across all hydrogel groups compared with the control. By day 3, both the LDHs/GelMA and MnO_2_@LDHs/GelMA groups exhibited a significant increase in cell viability. These findings indicate that all prepared hydrogels possess favorable biocompatibility, while the nanocomposite hydrogels further promote the proliferation of the tested cell types.

**Fig. 3:**
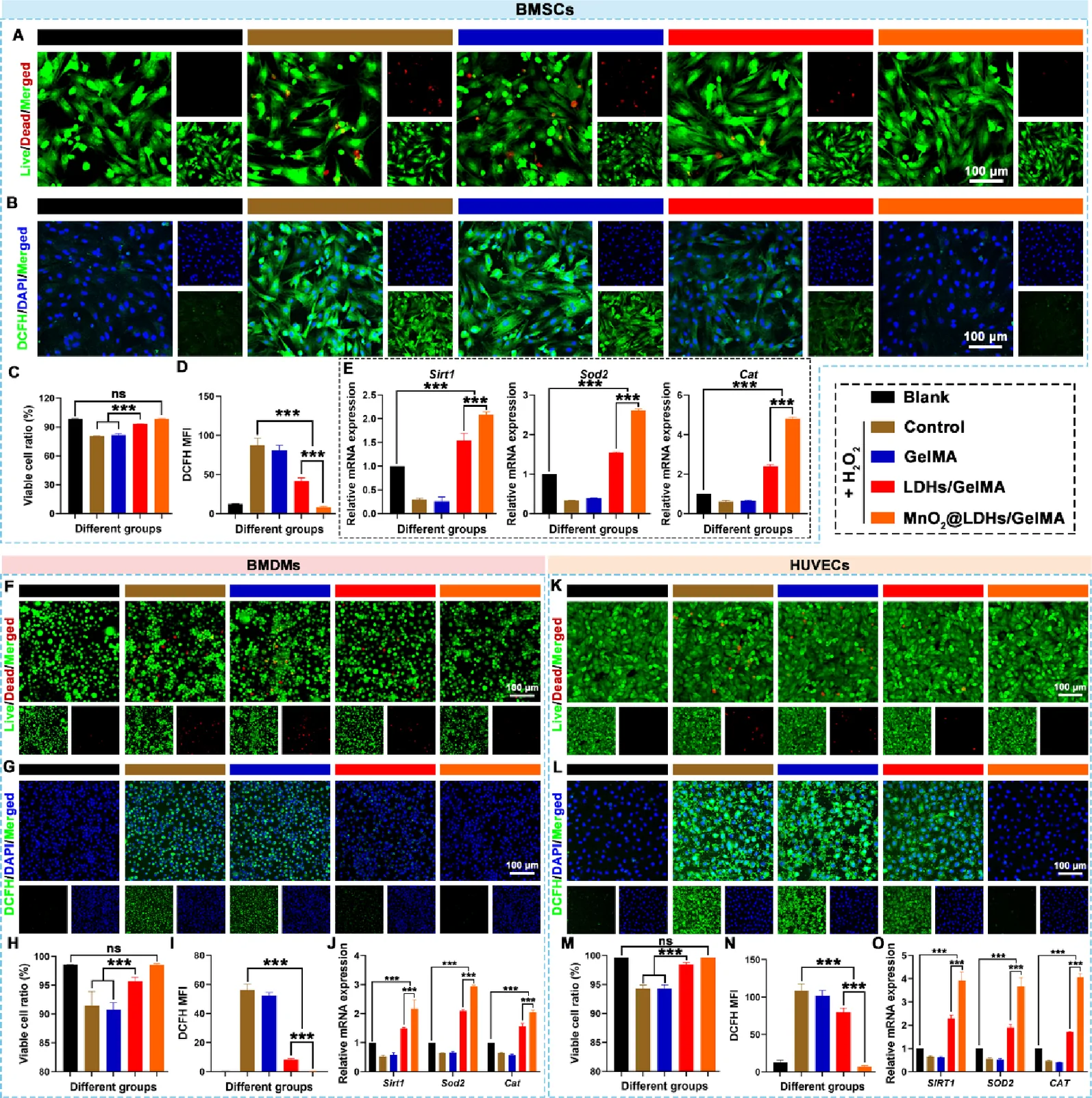
In vitro ROS-scavenging capacity and intracellular antioxidant protection of the hydrogels. **(A**,** C**,** F**,** H**,** K**,** M)** Live/Dead fluorescence staining and quantitative analysis of **(A**,** C)** BMSCs, **(F**,** H)** BMDMs, and **(K**,** M)** HUVECs under H_2_O_2_-induced oxidative stress (*n* = 4). **(B**,** D**,** G**,** I**,** L**,** N)** DCFH-DA fluorescence images for intracellular ROS detection and quantitative analysis of **(B**,** D)** BMSCs, **(G**,** I)** BMDMs, and **(L**,** N)** HUVECs under H_2_O_2_-induced oxidative stress (*n* = 4). **(E**,** J**,** O)** qPCR analysis of endogenous antioxidant genes in **(E)** BMSCs, **(J)** BMDMs, and **(O)** HUVECs (*n* = 4)

The pathological osteoporotic microenvironment is characterized by excessive ROS accumulation and oxidative stress, which compromise the viability and function of local cells [12]. As previous studies have demonstrated, H_2_O_2_ is the predominant ROS generated during bone injury and repair [22, 23]. Therefore, three key cell types involved in bone remodeling (BMSCs, BMDMs, and HUVECs) were subjected to a 100 µM H_2_O_2_ challenge in vitro to evaluate the cytoprotective capacity of the prepared hydrogels. Cell viability was assessed using Live/Dead fluorescence staining (Fig. 3A, F, and K) and corresponding quantitative analyses (Fig. 3C, H, and M). In the Control and GelMA groups, a high proportion of dead cells (red fluorescence) was observed across all three cell types, resulting in viable cell ratios significantly lower than those in the untreated Blank group. The LDHs/GelMA group exhibited partial improvement in cell survival. In contrast, predominantly viable cells (green fluorescence) were observed in the MnO_2_@LDHs/GelMA group, with viable cell ratios maintained at levels comparable with those of the Blank group. These results indicate that the nanocomposite hydrogel protects against oxidative stress-induced cell death.

To further investigate the mechanism underlying this cytoprotective effect, we assessed intracellular ROS-scavenging efficiency together with endogenous antioxidant responses. Intracellular ROS levels were evaluated using the DCFH-DA fluorescent probe (Fig. 3B, G, and L). Following H_2_O_2_ exposure, prominent green fluorescence was detected in the Control and GelMA groups for BMSCs, BMDMs, and HUVECs. Quantitative analysis of the fluorescence intensity (Fig. 3D, I, and N) showed a reduction in the LDHs/GelMA group compared with the GelMA group. The MnO_2_@LDHs/GelMA group displayed the lowest ROS accumulation among all H_2_O_2_-treated groups, reducing the fluorescence intensity to near-baseline levels. Furthermore, the mRNA expression levels of key endogenous antioxidant genes (*Sirt1*, *Sod2*, and *Cat*) were quantified via qPCR (Fig. 3E, J, and O). Across all three cell types, the transcript levels of these genes were significantly upregulated in the MnO_2_@LDHs/GelMA group compared with other groups. Collectively, these findings demonstrate that the MnO_2_@LDHs/GelMA hydrogel attenuates intracellular ROS accumulation while enhancing endogenous cellular antioxidant defenses, thereby alleviating ROS-induced oxidative stress in vitro.

### Bidirectional regulation of osteogenesis and osteoclastogenesis

**Fig. 4:**
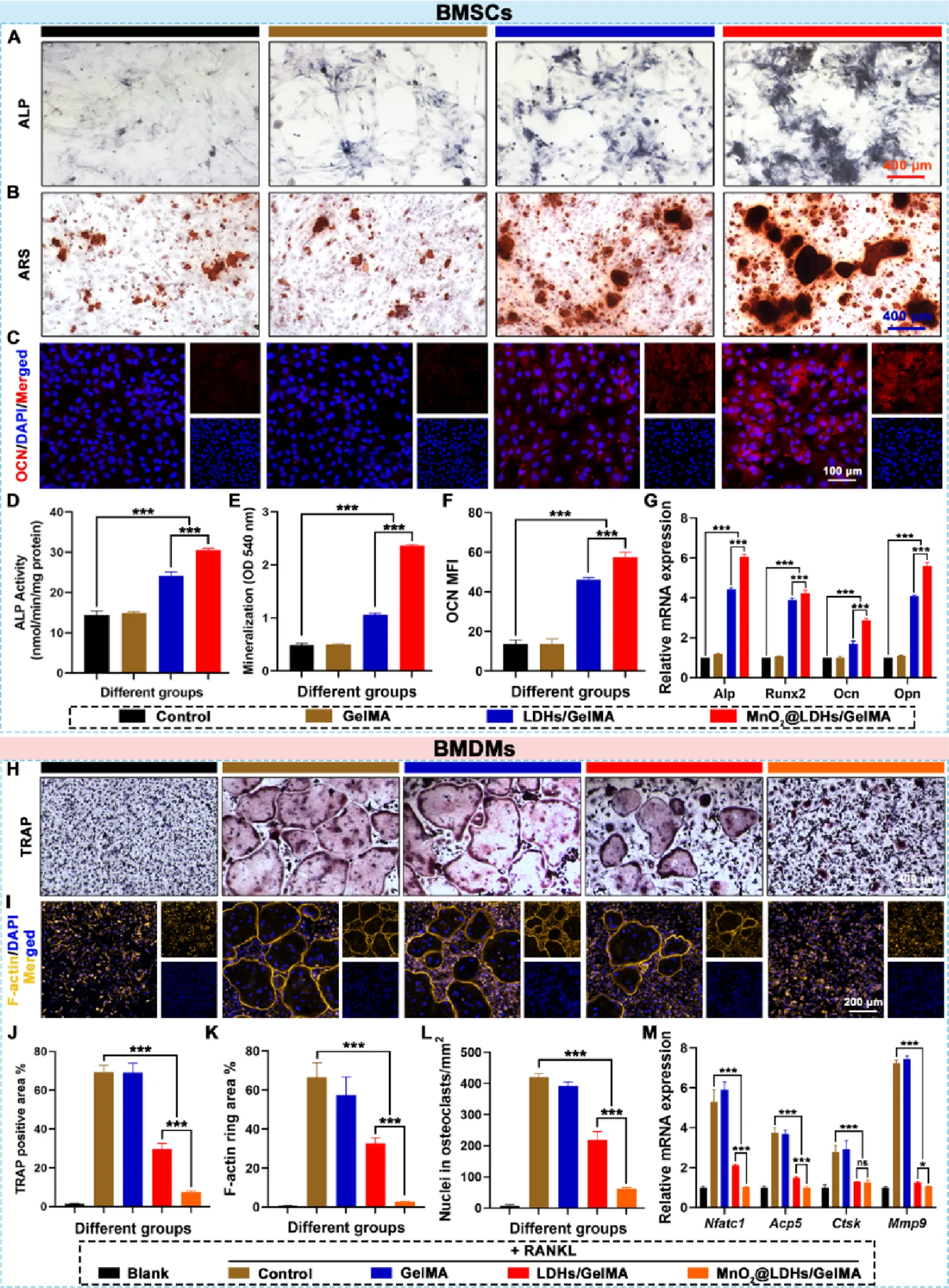
Bidirectional regulation of osteogenic differentiation and osteoclastogenesis in vitro. **(A)** ALP staining and **(D)** ALP activity quantification of BMSCs (*n* = 4). **(B)** ARS staining and **(E)** quantification of matrix mineralization of BMSCs (*n* = 4). **(C)** OCN immunofluorescence staining and **(F)** quantitative intensity analysis of BMSCs (*n* = 4). **(G)** qPCR analysis of osteogenic marker genes in BMSCs (*n* = 4). **(H)** TRAP staining and **(J)** TRAP-positive area quantification of RANKL-induced BMDMs (*n* = 4). **(I)** F-actin ring immunofluorescence staining of RANKL-induced BMDMs. **(K)** F-actin ring area and **(L)** nuclei number per cell quantification of BMDMs (*n* = 4). **(M)** qPCR analysis of osteoclastic marker genes in BMDMs (*n* = 4)

To evaluate the osteoinductive capacity of the prepared hydrogels, early osteogenic differentiation was assessed by alkaline phosphatase (ALP) staining and ALP activity quantification (Fig. 4A and D). To distinguish the biological effects of released ionic components from those of direct nanoparticle contact, BMSCs were first cultured solely in the hydrogel leachates. In this leachate-based culture model, both the LDHs/GelMA and MnO_2_@LDHs/GelMA groups demonstrated significantly enhanced ALP activity compared to the Control and GelMA groups (Fig. S6). Notably, there was no significant difference in osteogenic enhancement between the LDHs/GelMA and MnO_2_@LDHs/GelMA leachates. This equivalent performance indicates the independent osteoinductive capacity of the LDH framework, which is primarily driven by the sustained release of bioactive ions (e.g., Mg²⁺), and further suggests that the hydrogel leachates did not contain solid-phase MnO_2_ nanozymes. However, under direct contact culture conditions, a distinct model-dependent behavior emerged. Compared with the GelMA hydrogel, the LDHs/GelMA group exhibited elevated ALP activity, consistent with the inherent bioactivity of the LDH framework [20]. In contrast, during this direct contact, the MnO_2_@LDHs/GelMA group exhibited the most prominent positive staining and the highest ALP activity. For late-stage matrix mineralization, Alizarin Red S (ARS) staining showed that the LDHs/GelMA hydrogel partially enhanced matrix mineralization, whereas the MnO_2_@LDHs/GelMA group exhibited abundant, interconnected mineralized nodules, indicating markedly enhanced mineralization (Fig. 4B and E).

To further corroborate these findings at the molecular level, immunofluorescence and qPCR analyses were performed. OCN, a mature osteogenic marker, exhibited the highest immunofluorescence intensity in the MnO_2_@LDHs/GelMA group (Fig. 4C and F). Consistently, qPCR analysis revealed a significant upregulation of key osteogenic genes (*Alp*, *Runx2*, *Ocn*, and *Opn*) in the MnO_2_@LDHs/GelMA group compared with other groups (Fig. 4G). These data demonstrate that the MnO_2_@LDHs/GelMA hydrogel promotes osteogenic differentiation and matrix maturation.

Delayed healing of OP-BDs is closely associated with excessive osteoclastogenesis, a process in which intracellular ROS serve as key secondary messengers in the receptor activator of nuclear factor-κB ligand (RANKL) signaling pathway [29]. Therefore, we investigated the inhibitory effects of the hydrogels on RANKL-induced osteoclast differentiation in BMDMs. Tartrate-resistant acid phosphatase (TRAP) staining revealed the formation of numerous TRAP-positive multinucleated cells in both the Control and GelMA groups (Fig. 4H and J). The LDHs/GelMA group displayed a partial reduction in osteoclast formation. In contrast, the MnO_2_@LDHs/GelMA scaffold significantly reduced both the size and percentage area of these cells.

The assembly of the F-actin ring provides the structural foundation for osteoclasts to execute bone resorption [1, 20]. Immunofluorescence analyses of the F-actin cytoskeleton showed that while the LDHs/GelMA hydrogel partially impaired cytoskeletal reorganization, F-actin ring formation was markedly disrupted and the number of nuclei per osteoclast was significantly reduced by the MnO_2_@LDHs/GelMA hydrogel (Fig. 4I, K, and L). Furthermore, at the transcriptional level, the expression of key osteoclastogenic transcription factor (*Nfatc1*) and functional genes (*Acp5*, *Ctsk*, *Mmp9*) was downregulated in the MnO_2_@LDHs/GelMA group (Fig. 4M). Taken together, these findings indicate that the MnO_2_@LDHs/GelMA hydrogel suppresses RANKL-induced osteoclastogenesis and osteoclast function, thereby achieving bidirectional regulation of bone remodeling in vitro.

### Osteoimmune microenvironment remodeling and paracrine-mediated angiogenesis

**Fig. 5:**
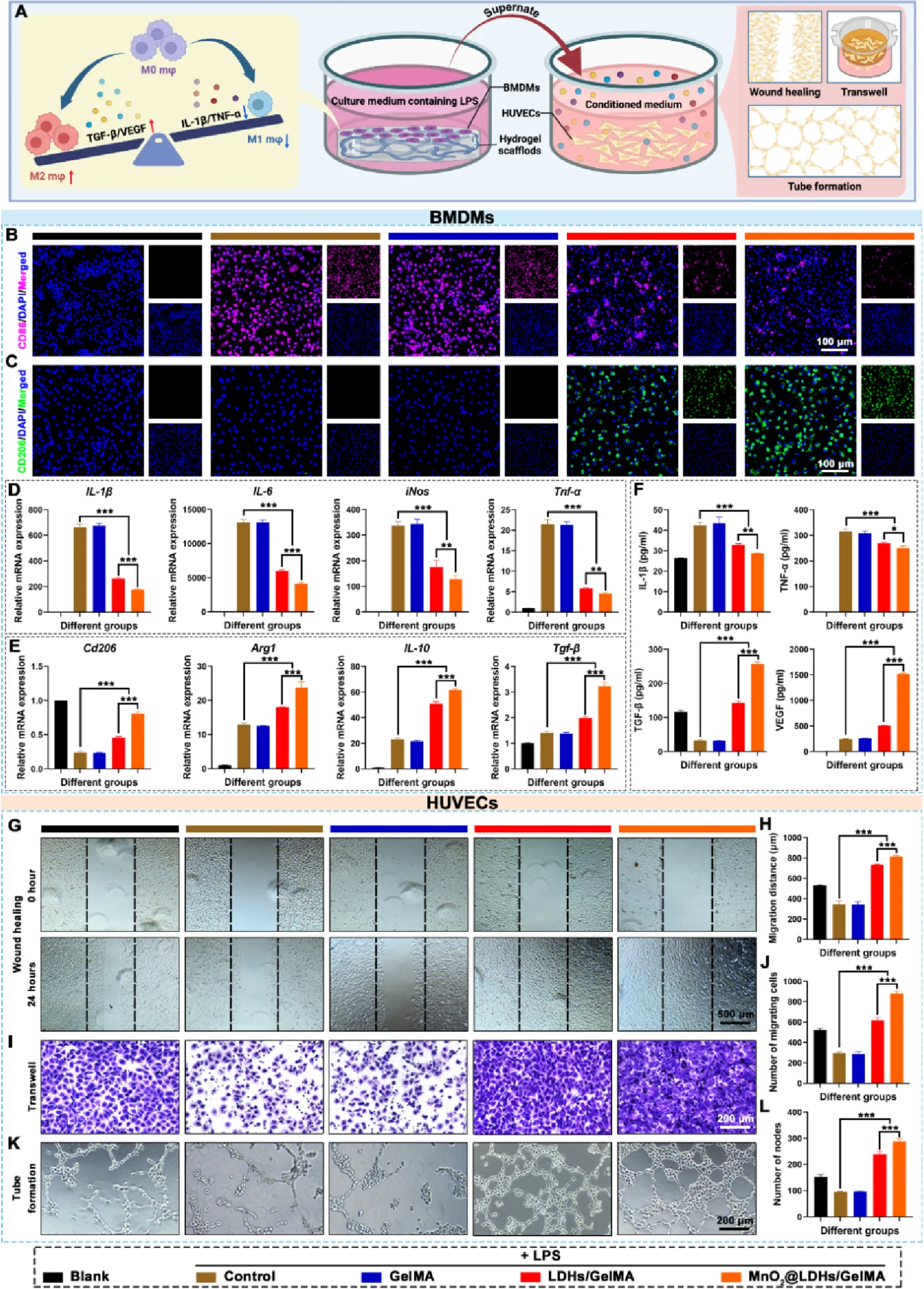
Macrophage polarization and paracrine-mediated angiogenesis evaluation in vitro. **(A)** Schematic illustration of the experimental design. Immunofluorescence staining of **(B)** CD86 (M1) and **(C)** CD206 (M2) markers in LPS-stimulated BMDMs. qPCR analysis of **(D)** M1-associated genes and **(E)** M2-associated genes in LPS-stimulated BMDMs (*n* = 4). **(F)** ELISA quantification of IL-1β, TNF-α, TGF-β, and VEGF secretion from LPS-stimulated BMDMs (*n* = 4). **(G)** Scratch wound healing images and **(H)** quantitative analysis of HUVECs treated with conditioned media (*n* = 4). **(I)** Transwell migration images and **(J)** quantitative analysis of HUVECs treated with conditioned media (*n* = 4). **(K)** Matrigel tube formation images and **(L)** quantitative analysis of HUVECs treated with conditioned media (*n* = 4)

In OP-BDs, chronic inflammation maintains macrophages in a pro-inflammatory M1 phenotype, which impairs the subsequent tissue repair cascade [30]. To evaluate the immunomodulatory capacity of the hydrogels, BMDMs were cultured under lipopolysaccharide (LPS)-induced inflammatory conditions (Fig. 5A). Immunofluorescence staining revealed that the Control and GelMA groups exhibited high expression of the M1 marker CD86, alongside minimal signals for the M2 marker CD206 (Fig. 5B and C). The LDHs/GelMA group induced a partial phenotypic shift, evidenced by a significant decrease in CD86 and a corresponding increase in CD206. The MnO_2_@LDHs/GelMA group displayed minimal CD86 expression while exhibiting the highest CD206 fluorescence intensity. These phenotypic observations were corroborated at the transcriptional level by qPCR (Fig. 5D and E), which confirmed that the MnO_2_@LDHs/GelMA hydrogel significantly downregulated M1-associated genes (*Il-1β*, *Il-6*, *iNos*, *Tnf-α*) and upregulated M2-associated genes (*Cd206*, *Arg1*, *Il-10*, *Tgf-β*). Furthermore, ELISA results showed a significant reduction in the secretion of inflammatory cytokines (IL-1β and TNF-α), coupled with a significant elevation in reparative and angiogenic factors (TGF-β and VEGF) in the MnO_2_@LDHs/GelMA group (Fig. 5F). These data demonstrate that the hydrogel modulates macrophage polarization toward a pro-regenerative phenotype, thereby suppressing the pathological inflammatory microenvironment.

Bone regeneration relies on the timely re-establishment of functional vascular networks, a process regulated by immune cell-derived paracrine signals [3]. To investigate this osteoimmune-angiogenic coupling, HUVECs were cultured using conditioned media derived from the aforementioned pre-treated BMDMs. Scratch wound healing and Transwell migration assays demonstrated that the M1-dominant conditioned media from the Control and GelMA groups severely impaired both horizontal and vertical migration of HUVECs (Fig. 5G–J). Conditioned media from the LDHs/GelMA group partially restored endothelial mobility. In contrast, the conditioned media from the MnO_2_@LDHs/GelMA group induced the highest migration and chemotactic recruitment. The functional angiogenic capacity was further assessed via a Matrigel tube formation assay (Fig. 5K and L). HUVECs treated with the MnO_2_@LDHs/GelMA-conditioned media self-assembled into well-connected capillary-like networks, exhibiting a significantly greater number of branch nodes compared with the other groups. These findings suggest that the MnO_2_@LDHs/GelMA hydrogel-modulated osteoimmune microenvironment creates a favorable paracrine cascade, thereby promoting endothelial cell migration and microvascular network formation.

### *In vivo* repair of OP-BDs

Prior to evaluating bone repair, the osteoporotic rat model was successfully established via OVX and confirmed by comprehensive radiological and serum evaluations (Fig. S7A-D). Furthermore, post-implantation histological and biochemical profiling confirmed the in vivo biosafety of the hydrogels, with no evidence of systemic toxicity or major organ abnormalities across all groups (Fig. S7E-G).

**Fig. 6:**
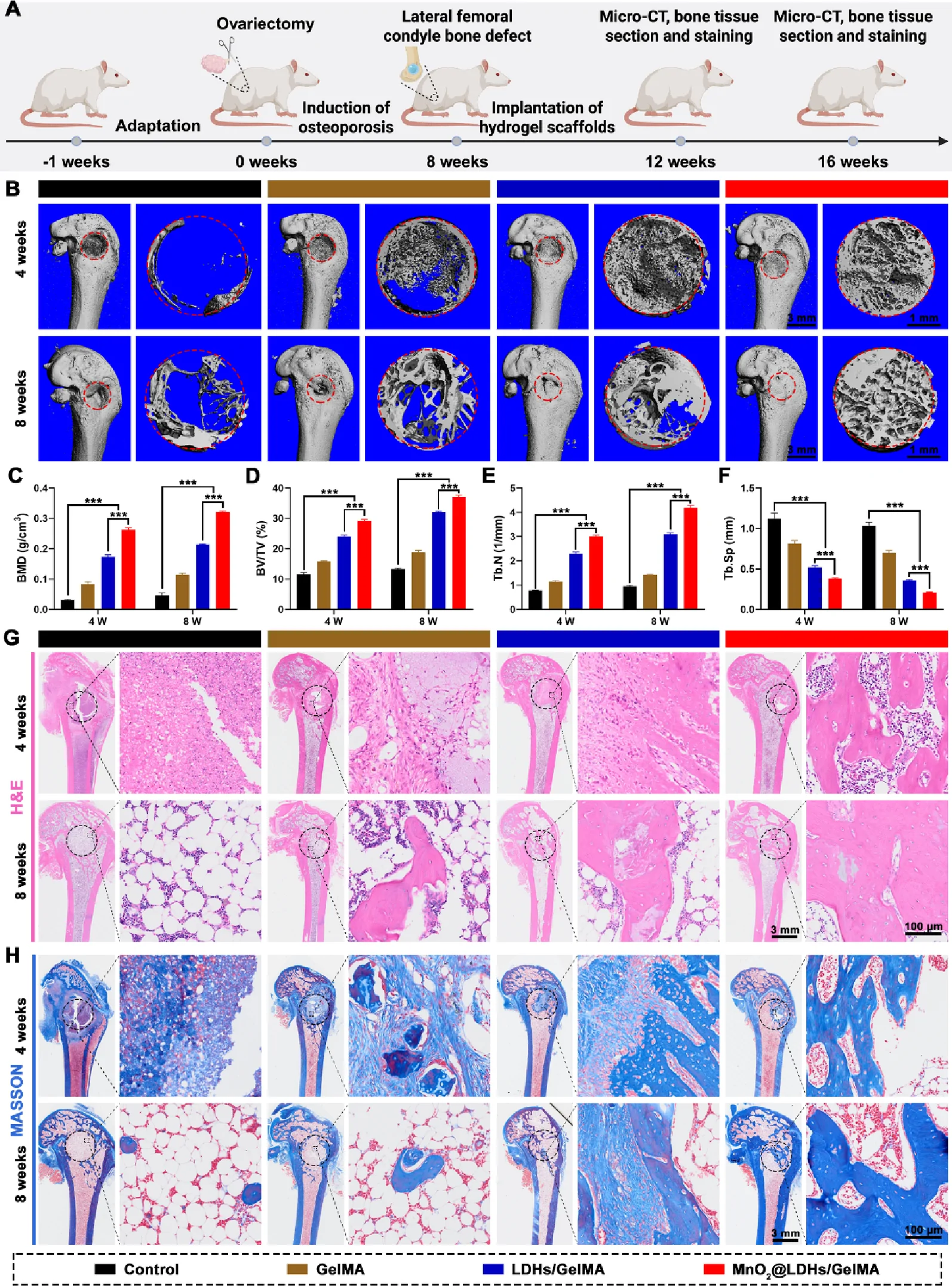
Radiological and histomorphological evaluation of OP-BDs repair in vivo. **(A)** Schematic illustration of the experimental design. **(B)** 3D micro-CT images of lateral femoral condyle defects at 4 and 8 weeks post-implantation. Quantitative morphometric analysis of **(C)** BMD, **(D)** BV/TV, **(E)** Tb.N, and **(F)** Tb.Sp within the defect regions (*n* = 4). **(G)** H&E staining and **(H)** Masson’s trichrome staining images of the defect regions at 4 and 8 weeks post-implantation

Critical-sized lateral femoral condyle defects were established to evaluate the in vivo bone regenerative efficacy of the hydrogels (Fig. 6A). At 4 weeks post-implantation, micro-CT 3D reconstructions revealed minimal new bone formation in the Control and GelMA groups, with the defect cavities remaining largely unoccupied (Fig. 6B). The LDHs/GelMA group showed preliminary reticular woven bone. In contrast, the MnO_2_@LDHs/GelMA group exhibited abundant newly formed bone. By week 8, the Control and GelMA groups still displayed unhealed defects, and the LDHs/GelMA group exhibited only partial defect bridging. In contrast, the MnO_2_@LDHs/GelMA group exhibited complete defect bridging. Quantitative morphometric analyses (Fig. 6C–F) confirmed that the MnO_2_@LDHs/GelMA hydrogel yielded the highest bone mineral density (BMD), bone volume fraction (BV/TV), and trabecular number (Tb.N), alongside the lowest trabecular separation (Tb.Sp). These radiological results indicate that the MnO_2_@LDHs/GelMA hydrogel accelerates new bone regeneration and structural bridging in osteoporotic defects.

To further corroborate these radiological findings at the tissue and cellular levels, histological evaluations via H&E and Masson’s trichrome staining were conducted. At week 4, the LDHs/GelMA group displayed moderate collagen deposition and fragmented osteoid formation. Conversely, the MnO_2_@LDHs/GelMA group exhibited continuous, eosinophilic newly formed bone matrices populated with numerous osteocytes (Fig. 6G), alongside dense blue-stained collagen networks providing a template for mineralization (Fig. 6H). By week 8, the LDHs/GelMA group exhibited a mixture of loose woven bone and limited nascent lamellar bone. In contrast, the MnO_2_@LDHs/GelMA group exhibited complete defect bridging by ordered and mineralized mature lamellar bone that was structurally integrated with the host tissue. These histological findings confirm that the MnO_2_@LDHs/GelMA hydrogel facilitates the transition from early woven bone to mature lamellar bone.

### *In vivo* multidimensional microenvironment remodeling: osteogenesis, osteoclastogenesis, immunity, and angiogenesis

**Fig. 7:**
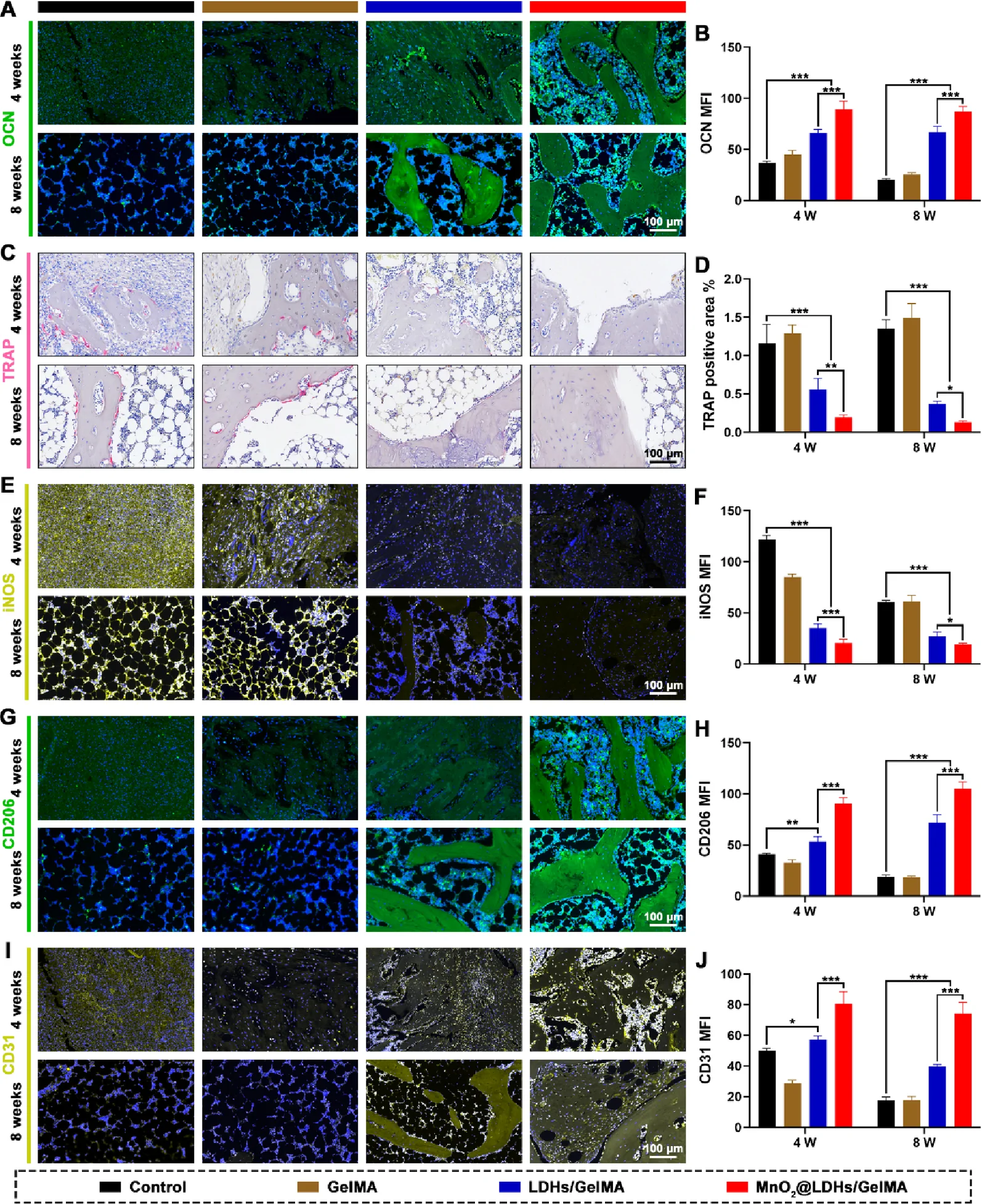
Evaluation of osteogenesis, osteoclastogenesis, macrophage polarization, and angiogenesis in vivo. **(A)** OCN immunofluorescence staining and **(B)** quantitative intensity analysis within the defect area at 4 and 8 weeks (*n* = 4). **(C)** TRAP staining and **(D)** quantitative analysis of osteoclasts within the defect area (*n* = 4). In situ immunofluorescence staining of **(E)** iNOS and **(G)** CD206 for macrophage polarization evaluation. Quantitative intensity analysis of **(F)** iNOS and **(H)** CD206 (*n* = 4). **(I)** CD31 immunofluorescence staining and **(J)** quantitative analysis of vascularization (*n* = 4)

The core pathology of OP-BDs lies in an imbalance of bone remodeling, where osteoclast-mediated bone resorption outpaces osteoblast-mediated bone formation [11, 12]. To evaluate whether the hydrogels could reverse this metabolic uncoupling in vivo, in situ osteoblast and osteoclast activities within the defect regions were assessed. Immunofluorescence for OCN showed minimal fluorescence in the Control and GelMA groups (Fig. 7A and B). The LDHs/GelMA group exhibited a moderate increase in OCN expression, whereas the MnO_2_@LDHs/GelMA group exhibited the most prominent and widely distributed OCN expression. Conversely, TRAP staining demonstrated elevated pathological osteoclastogenesis (characterized by numerous multinucleated cells) in the Control and GelMA groups. While the LDHs/GelMA group partially reduced the formation of these resorptive cells, the pathological osteoclastogenesis was markedly suppressed in the MnO_2_@LDHs/GelMA group (Fig. 7C and D). These data indicate that the MnO_2_@LDHs/GelMA hydrogel re-establishes the physiological balance between osteogenesis and osteoclastogenesis in vivo.

The success of in vivo bone healing is regulated by the local immune microenvironment, where excessive ROS accumulation sustains a pro-inflammatory macrophage phenotype and delays tissue repair [30]. To determine the in situ immunomodulatory effects of the implants, macrophage polarization within the defect niche was evaluated. Immunofluorescence demonstrated that the expression of iNOS (M1 marker) was prominent in the Control and GelMA groups. The LDHs/GelMA group partially shifted macrophage polarization, as evidenced by moderately decreased iNOS and elevated CD206 signals. In the MnO_2_@LDHs/GelMA group, iNOS expression was significantly downregulated (Fig. 7E and F), while the expression of CD206 increased, exhibiting the highest fluorescence intensity among all groups (Fig. 7G and H). These findings demonstrate that the nanocomposite modulates macrophage polarization toward a pro-regenerative phenotype, thereby attenuating the pathological inflammatory microenvironment in the osteoporotic defect.

Bone regeneration depends on the timely re-establishment of functional vascular networks, which are impaired by excessive ROS accumulation and persistent inflammation in osteoporotic defects [3]. Finally, to investigate the in vivo angiogenic response, CD31 immunofluorescence was performed (Fig. 7I and J). The results revealed that the osteoporotic ischemic state severely impaired angiogenesis in the untreated Control and GelMA groups. Consistent with its moderate immunomodulatory effects, the LDHs/GelMA group promoted partial vascularization. However, the MnO_2_@LDHs/GelMA scaffold promoted the formation of dense, interconnected capillary networks as early as week 4, maintaining vascularization through week 8. These findings suggest that the MnO_2_@LDHs/GelMA hydrogel-modulated osteoimmune microenvironment promotes angiogenesis–osteogenesis coupling, thereby facilitating bone regeneration.

## Discussion

The core pathology of OP-BDs is characterized by a severe imbalance in bone remodeling, driven by chronic oxidative stress and persistent inflammation, which collectively uncouple osteoclast-mediated bone resorption from osteoblast-mediated bone formation [9–12]. Traditional bone tissue engineering strategies, which primarily rely on providing passive physical support or single osteogenic cues, often exhibit limited therapeutic efficacy in this ROS-enriched milieu [13]. In this study, we developed a microenvironment-remodeling nanocomposite hydrogel (MnO_2_@LDHs/GelMA) to counteract these pathological processes. By integrating the catalytic ROS-scavenging activity of the MnO_2_ nanozyme with the intrinsic structural bioactivity of the LDH framework, this strategy not only protects resident cells from oxidative damage but also facilitates a multidimensional repair cascade, including the modulation of macrophage polarization toward a pro-regenerative phenotype, the promotion of paracrine-mediated angiogenesis, and the restoration of bone remodeling homeostasis.

Our crystallographic and catalytic evaluations revealed that the in situ grown MnO_2_ layer was amorphous. This amorphous structure provides abundant surface defect sites that serve as catalytic centers for the catalase-like activity [31–34]. As demonstrated by the rapid H_2_O_2_ degradation and continuous O_2_ generation observed in our quantitative assays, this catalytic property enables the nanocomposite to efficiently scavenge excess ROS and thereby remodel the oxidative microenvironment while alleviating local hypoxia. Furthermore, the morphological stability observed in our degradation assays confirms that the surface-deposited MnO_2_ layer acts as both an active chemical shield and a physical barrier [35]. By catalytically decomposing H_2_O_2_ and consuming local protons, the MnO_2_ coating protects the underlying basic LDH framework from acid-induced degradation commonly encountered in the inflammatory osteoporotic niche, thereby ensuring the sustained release of bioactive cues [36].

Upon incorporation of the nanoparticles into the GelMA matrix, we observed changes in the mechanical properties that guided the optimization of the hydrogel formulation. The introduction of LDHs resulted in a slight decrease in the mechanical strength of the hydrogel. We attribute this to the UV-shielding effect of the opaque LDH nanosheets, which impaired photoinitiator activation during the crosslinking process [24, 37, 38]. At higher nanoparticle loading (1%), this UV-shielding effect, together with the increased matrix stiffness, also explains the reduced cellular metabolic activity and disrupted actin organization observed in our in vitro experiments. Conversely, the optimized 0.25% (w/v) MnO_2_@LDHs/GelMA composite demonstrated improved mechanical performance. Consistent with the red-shift observed in the FTIR spectra, this nano-reinforcement effect is driven by the formation of a stable hydrogen-bonding network between the active polar groups of MnO_2_@LDHs and the GelMA polymer chains [39]. The homogeneously dispersed nanoparticles act as additional physical crosslinking sites, increasing the sliding resistance of the polymer chains and thereby enhancing the macroscopic stiffness of the hydrogel [40, 41]. Consequently, this reinforced physical crosslinking and nano-reinforcement effect also endows the MnO_2_@LDHs/GelMA hydrogel with superior mechanical stability during hydrolytic degradation. As evidenced by the dynamic monitoring of the compressive modulus in PBS, the nanocomposite hydrogel effectively resisted rapid mechanical deterioration, retaining a robust modulus throughout the 14-day observation period. This sustained mechanical support is expected to facilitate continuous cell infiltration and subsequent bone ingrowth within the defect site. Concurrently, the hydrogel maintains a degradation profile (~6 weeks) that parallels the in vivo window of woven bone formation [42–44].

The catalase-like activity of the surface-decorated MnO_2_ catalyzes the decomposition of accumulated H_2_O_2_ into H_2_O and in situ-generated O_2_ [23]. This conversion not only reduces the extracellular ROS burden but also alleviates the local hypoxia associated with ischemic bone defects. Consequently, the alleviated oxidative microenvironment activates endogenous antioxidant signaling pathways, as evidenced by the upregulation of *Sirt1*, *Sod2*, and *Cat*, thereby strengthening cellular antioxidant defenses and ensuring the survival of BMSCs, BMDMs, and HUVECs under oxidative stress [45, 46].

Beyond acute cellular protection, remodeling the osteoimmune microenvironment plays a pivotal role in long-term tissue regeneration [30]. Our previous studies established that nitrate-intercalated, strontium-doped LDHs serve as a bioactive nanoplatform that intrinsically suppresses pro-inflammatory gene expression [18–20]. Consistent with this, the pure LDHs/GelMA group exhibited a basal anti-inflammatory capacity. However, the MnO_2_@LDHs/GelMA nanocomposite demonstrated synergistic immunomodulatory effects. The structural nitrate ions within the LDHs modulate macrophage polarization at the transcriptional level, whereas chronic oxidative stress is known to impair the acquisition of a pro-regenerative macrophage phenotype [12, 30]. By scavenging ROS, the MnO_2_ nanozyme re-establishes a microenvironment conducive to pro-regenerative macrophage polarization. Collectively, these complementary mechanisms establish a pro-regenerative osteoimmune microenvironment, in which pro-regenerative macrophages secrete bioactive factors (e.g., VEGF and TGF-β), thereby promoting endothelial network assembly through paracrine signaling [3].

Together with the ROS-depleted microenvironment, the nanocomposite bidirectionally regulates bone remodeling. For osteogenesis, our comparative in vitro models reveal a dual-stage mechanism. As evidenced by the leachate assays, the LDH framework serves as the primary chemical mediator of osteogenesis through the sustained release of bioactive ions (such as Mg^2+^), which intrinsically promote BMSC osteogenic differentiation [20]. However, the superior osteogenic performance of the MnO_2_@LDHs/GelMA hydrogel during direct contact culture highlights the importance of surface-confined nanozyme catalysis. By effectively scavenging endogenous ROS via the firmly anchored MnO_2_ nanozymes, the scaffold provides an in situ antioxidant shield that prevents ROS-induced osteoblast dysfunction, thereby creating a favorable microenvironment for ion-mediated osteogenesis. For osteoclastogenesis, intracellular ROS serve as key secondary messengers in the RANKL-induced signaling cascade driving osteoclast fusion and maturation [29]. By scavenging intracellular ROS via the MnO_2_ nanozyme, the scaffold attenuates downstream osteoclastogenic signaling, leading to the disruption of F-actin ring polarization observed in our in vitro assays [1, 20]. Ultimately, this dual action—supporting osteoblast function while suppressing osteoclastogenesis—contributes to the restoration of bone remodeling homeostasis.

 In vivo evaluations demonstrated that these molecular mechanisms translated into functional tissue repair. The pure LDHs/GelMA group exhibited limited long-term regenerative efficacy, characterized by a mixture of loose woven bone and only limited mature lamellar bone at week 8, reflecting the structural vulnerability of unprotected LDHs within the pathological osteoporotic microenvironment. In contrast, the MnO_2_ coating preserved the structural integrity of the LDHs by protecting them against acid-induced degradation within the pathological microenvironment. Accordingly, the MnO_2_@LDHs/GelMA hydrogel promoted the progression from early woven bone to mature lamellar bone, ultimately achieving complete defect closure and seamless structural integration with the host bone [42–44].

Taken together, the MnO_2_@LDHs/GelMA hydrogel mitigates the pathological progression of OP-BDs through MnO_2_-mediated ROS scavenging, thereby protecting the bioactive LDH framework and modulating macrophage polarization toward a pro-regenerative phenotype. This optimized osteoimmune microenvironment, together with the intrinsic bioactivity of the preserved LDH nanoplatform, coordinates paracrine-mediated angiogenesis and bone remodeling, ultimately promoting vascularized bone regeneration. Despite these promising results, we acknowledge that our contained metaphyseal defect model may not adequately represent mechanically unstable fractures or segmental defects, and that the current repair outcomes are primarily based on radiographic and histological evaluations. Therefore, future studies employing mechanically demanding large-animal models together with direct biomechanical testing are necessary to validate the long-term functional performance and degradation behavior of the hydrogel. Furthermore, leveraging multi-omics approaches to decipher the intricate cellular crosstalk within the remodeled niche will provide deeper mechanistic insights and facilitate the clinical translation of this multifunctional biomaterial platform.

## Conclusion

In conclusion, we developed a microenvironment-remodeling nanocomposite hydrogel (MnO_2_@LDHs/GelMA) to repair OP-BDs. By integrating the catalytic ROS-scavenging activity of the MnO_2_ nanozyme with the structural bioactivity of the protected LDH nanoplatform, the hydrogel neutralizes pathological oxidative stress (Scheme 1). This process remodels the osteoimmune microenvironment by modulating macrophage polarization toward a pro-regenerative phenotype, thereby promoting paracrine-mediated angiogenesis. The hydrogel further exerts bidirectional regulation of bone remodeling by promoting osteogenesis while suppressing RANKL-induced osteoclastogenesis. In vivo evaluations in an osteoporotic rat lateral femoral condyle defect model demonstrated that the hydrogel re-establishes bone remodeling homeostasis, thereby accelerating vascularized bone regeneration. Collectively, this work provides a promising translational biomaterial strategy for the treatment of OP-BDs and other complex metabolic bone diseases.

## Materials and methods

### Synthesis of SrMgAl-LDH-NO_3_^-^ (LDHs) nanosheets

SrMgAl-LDH-NO_3_^-^ (hereafter referred to as LDHs) nanosheets were synthesized via a hydrothermal-assisted coprecipitation and ion-exchange method as previously reported [20]. Briefly, the precursor salts Mg(NO_3_)_2_·6H_2_O and Al(NO_3_)_3_·9H_2_O were coprecipitated under alkaline conditions, followed by an ion-exchange process with Sr(NO_3_)_2_. The resulting suspension was then hydrothermally treated in a Teflon-lined autoclave at 140 °C for 36 h. The LDH nanosheets were collected by centrifugation and washed with deionized water.

### Preparation of MnO_2_@LDHs nanocomposites

LDHs (400 mg) were ultrasonically dispersed in deionized water. Under continuous stirring (1000 rpm) at 50 °C, the dispersion was added dropwise to 20 mL of KMnO_4_ solution (100 mM). The resulting mixture was allowed to react for 1 h to facilitate MnO_4_^-^ adsorption. After centrifugation and washing, the purplish-red intermediate was resuspended. Subsequently, 10 mL of absolute ethanol (≥ 99.7%) was added dropwise as the reducing agent under continuous stirring at 50 °C for 30 min until the solution turned brown [47]. The MnO_2_@LDHs nanocomposites were collected by centrifugation and washed with deionized water.

### Characterization and *in vivo* stability of nanoparticles

The morphology and elemental distribution of the nanoparticles were characterized by TEM (JEOL JEM-F200, Japan) equipped with an EDS detector. The crystal structure and surface chemical states were analyzed by XRD (Bruker D2, Germany), FTIR (Thermo Fisher Nicolet iS20, USA), and XPS (Thermo Fisher K-Alpha, USA). The elemental composition was quantified using ICP-MS (Agilent 7800, USA). To evaluate the catalase-like ROS-scavenging ability of the nanocomposites, a Ti(SO_4_)_2_ colorimetric assay was used to monitor H_2_O_2_ degradation kinetics. Furthermore, dissolved oxygen (DO) levels were measured using a DO meter to monitor real-time O_2_ generation during the H_2_O_2_ decomposition. To evaluate the protective effect of the MnO_2_ coating against acid-induced degradation in vivo, LDHs and MnO_2_@LDHs nanoparticles were implanted around the lateral femoral condyle defect sites of osteoporotic rats. The implants were retrieved at 7 and 14 days post-implantation, and their morphological stability and structural integrity were evaluated by TEM.

### Fabrication of composite hydrogels

The photoinitiator LAP (2.5 mg/mL) was dissolved in PBS. GelMA (EFL, China) was subsequently dissolved at 60 °C in the dark to prepare a 5% (w/v) GelMA pre-gel solution [24]. UV-sterilized LDHs and MnO_2_@LDHs nanoparticles were uniformly dispersed in the solution at a final concentration of 0.25% (w/v). The mixed precursor was injected into molds and crosslinked under 405 nm UV light irradiation for 30 s to form the LDHs/GelMA and MnO_2_@LDHs/GelMA composite hydrogels.

### Characterization and degradation of hydrogels

The internal porous structure and elemental distribution of the lyophilized hydrogels were characterized by SEM (ZEISS Gemini 300, Germany) equipped with EDS. The surface hydrophilicity of the hydrogels was evaluated using a contact angle goniometer (DataPhysics OCA20, Germany). The equilibrium swelling ratio was determined by immersing the gels in PBS at 37 °C for 24 h. Mechanical properties were evaluated using a universal testing machine (Instron 5943, USA) at a compression rate of 2 mm/min. To assess mechanical stability during degradation, the compressive Young’s modulus (at 25% strain) of hydrogels immersed in PBS (37 °C) was measured on days 1, 7, and 14. For the degradation assay, the hydrogels were evaluated in vitro in PBS at 37 °C and in vivo around the lateral femoral condyle defect sites of osteoporotic rats. The samples were retrieved and weighed at weeks 2, 4, and 6 to calculate the remaining mass percentage. To evaluate the in vitro ion release profiles, MnO_2_@LDHs/GelMA hydrogel sample (1 mL) was immersed in 40 mL of PBS and incubated at 37 °C under continuous shaking. At predetermined time points (days 1, 3, 5, 7, and 14), 10 mL of the release medium was collected and immediately replenished with an equal volume of fresh PBS. The released Mg^2+^ and Mn^2+^ concentrations were quantitatively analyzed using ICP-MS.

### Cell culture

Rat BMSCs, rat BMDMs, and HUVECs were isolated or purchased from standard cell banks. BMSCs and BMDMs were cultured in α-MEM supplemented with 10% FBS and 1% penicillin-streptomycin, whereas HUVECs were cultured in DMEM containing 10% FBS. All cells were maintained in a humidified incubator at 37 °C with 5% CO_2_, and the culture medium was refreshed every 2–3 days.

### Cytocompatibility assay

BMSCs, BMDMs, and HUVECs were seeded onto different hydrogels. After 1, 2, and 3 days of culture, CCK-8 reagent was added and incubated for 2 h. The absorbance at 450 nm was measured using a microplate reader to evaluate cell proliferation and metabolic activity.

### qPCR analysis

Total RNA was extracted from cells using TRIzol reagent and reverse-transcribed into cDNA using the PrimeScript^™^ RT reagent kit. Real-time qPCR was performed using SYBR Green qPCR Master Mix. Relative gene expression levels were calculated using the 2^-∆∆Ct^ method, with *Gapdh* serving as the endogenous reference gene. All primer sequences are listed in Table S1.

### Immunofluorescence staining

Cells were fixed with 4% paraformaldehyde, permeabilized with 0.1% Triton X-100, and blocked with 5% BSA. Subsequently, cells were incubated sequentially with primary antibodies and corresponding fluorophore-conjugated secondary antibodies. The primary antibodies used are listed in Table S2. Nuclei were counterstained with DAPI. Fluorescence images were acquired using a confocal laser scanning microscope (CLSM) and quantitatively analyzed using ImageJ software.

### *In vitro* intracellular ROS scavenging assay

Cells were seeded onto the hydrogels and treated with 100 µM H_2_O_2_ to simulate an oxidative stress microenvironment. After 24 h of treatment, cell viability was assessed using a Live/Dead assay kit, and samples were observed under CLSM to calculate the survival rates. For intracellular ROS detection, cells were incubated with 10 µM DCFH-DA probe in the dark for 20 min, followed by H_2_O_2_ stimulation for 2 h prior to CLSM imaging and fluorescence intensity quantification. Simultaneously, total RNA was extracted, and the expression levels of endogenous antioxidant genes (*Sirt1*, *Sod2*, *Cat*) were quantified by qPCR as described in Sect. "qPCR Analysis".

### *In vitro* osteogenic differentiation

 To evaluate the independent biological effects of the released ions, a leachate-induced model was established. Briefly, the crosslinked hydrogels were incubated in the basal culture medium at a volume-to-volume ratio of 100 µL/mL at 37 °C for 24 h. The supernatants were collected and sterilized through a 0.22 μm membrane filter, and supplemented with osteogenic induction factors to obtain conditioned leachates. BMSCs were either seeded directly onto the hydrogels for direct contact culture or cultured with the prepared leachates in an osteogenic induction medium as previously described [20, 21]. Gene expression (*Alp*, *Runx2*, *Ocn*, *Opn*) was analyzed by qPCR on day 4 as described in Sect. "qPCR Analysis". ALP staining and activity assays were performed on day 7, and ALP activity was normalized to total intracellular protein content measured using a BCA assay kit to eliminate bias from variations in cell number. Immunofluorescence staining of OCN was conducted on day 14 following the protocol described in Sect. "Immunofluorescence Staining". On day 21, extracellular matrix mineralization was evaluated by ARS staining, and calcium deposition was quantified after dissolution in 10% cetylpyridinium chloride.

### *In vitro* osteoclastogenesis

BMDMs were seeded onto the hydrogels and induced to differentiate into osteoclasts using a medium supplemented with 100 ng/mL RANKL and 40 ng/mL M-CSF. After 5–7 days of culture, the cells were stained using a TRAP staining kit, and the area and number of multinucleated osteoclasts were quantified. The cytoskeleton was stained with FITC-phalloidin to visualize the integrity of the F-actin rings in osteoclasts. Meanwhile, the expression of osteoclast-specific genes (*Nfatc1*, *Acp5*, *Ctsk*, *Mmp9*) was analyzed by qPCR as described in Sect. "qPCR Analysis".

### Macrophage polarization assay

An inflammatory microenvironment was established in BMDMs by stimulation with 200 ng/mL LPS. After 24 h of culture, cells were fixed and permeabilized, and in situ immunofluorescence staining was performed using antibodies to evaluate the expression of the M1 marker CD86 and M2 marker CD206. Cellular RNA was extracted, and the expression levels of pro-inflammatory genes (*Il-1β*, *Il-6*, *iNos*, *Tnf-α*) and anti-inflammatory genes (*Cd206*, *Arg1*, *Il-10*, *Tgf-β*) were analyzed by qPCR as described in Sect. "qPCR Analysis". Additionally, the levels of secreted factors (IL-1β, TNF-α, TGF-β, VEGF) in the LPS-induced inflammatory microenvironment were quantified using ELISA kits.

### Paracrine-mediated angiogenesis assays

The culture supernatants of BMDMs after 24 h of LPS stimulation were collected and centrifuged to obtain conditioned media (CM). Subsequently, HUVECs were cultured with CM to evaluate paracrine angiogenic effects: (1) a scratch wound healing assay was performed to evaluate the horizontal migration ability of HUVECs after 24 h of treatment; (2) a Transwell migration assay was performed to assess the vertical migratory ability of HUVECs after 12 h of incubation; (3) a Matrigel tube formation assay was performed to evaluate the ability of HUVECs to form capillary-like networks after 4–6 h of incubation on Matrigel.

### Establishment of OP model and surgical procedure

Eight-week-old female SD rats were used to establish the OP model via OVX, with a sham-operated group (Sham) serving as control [48]. Eight weeks after surgery, successful establishment of the OP model was confirmed by micro-CT analysis, H&E staining, and serum E2 level measurement. Subsequently, a critical-sized bone defect (3 mm in diameter and 3 mm in depth) was created at the lateral femoral condyle using a 3 mm diameter dental trephine bur. The prefabricated hydrogels from each group were implanted into the defect sites, followed by layer-by-layer suturing.

### Micro-CT analysis

At 4 and 8 weeks post-implantation, rats were euthanized and femoral specimens were harvested. Bone defect regions were scanned using a high-resolution micro-CT system (µCT 35, Scanco Medical AG, Switzerland). To ensure high reproducibility and accurate quantitative analysis, the scanning parameters were strictly set as follows: an X-ray voltage of 65 kV, a current of 385 µA, a voxel size of 10 μm, and a 1.0 mm Al filter to reduce beam-hardening artifacts.

3D images were reconstructed using the built-in software. During image processing, a 3D Gaussian filter (sigma = 0.8, support = 1) was applied to reduce image noise. A global grayscale threshold of 100 was used to distinguish mineralized bone from non-mineralized tissues, thereby minimizing radiodensity interference from soft tissues and residual hydrogel during quantitative analysis of new bone formation. Quantitative analyses of the BMD, BV/TV, Tb.N, and Tb.Sp were performed within a standardized cylindrical volume of interest (VOI; diameter 3.0 mm and height 2.0 mm) centered coaxially on the original defect site.

### Histological, immunofluorescence, and biosafety evaluations

The harvested femoral specimens were decalcified using EDTA, embedded in paraffin, and sectioned into 5 μm serial slices. H&E and Masson’s trichrome staining were performed to evaluate new bone formation and collagen deposition, while TRAP staining was used to assess osteoclast presence in vivo. Immunofluorescence staining of the tissue sections was conducted to evaluate osteogenesis (OCN), macrophage polarization (iNOS/CD206), and angiogenesis (CD31). In addition, whole blood and serum samples were collected 8 weeks post-surgery for routine hematological analysis and biochemical assays (ALT, AST, CREA, UA). Major organs (heart, liver, spleen, lung, kidney) were subjected to H&E staining to assess systemic biosafety.

### Statistical analysis

All quantitative data were analyzed using GraphPad Prism (version 9.5.1) and are presented as the mean ± standard deviation. Comparisons between two groups were performed using an unpaired Student’s t-test. One-way analysis of variance (ANOVA) followed by Tukey’s post hoc test was used for multiple-group comparisons. Statistical significance was defined as *p < 0.05. Significance levels are indicated as *p < 0.05, **p < 0.01, and ***p < 0.001; ns indicates no significant difference.

## Supplementary Information

Supplementary Material 1

## Data Availability

No datasets were generated or analysed during the current study.
