# Supplementary material for: Layered double hydroxide-based ROS-scavenging nanozyme hydrogels re-establish osteoimmune-angiogenic coupling for osteoporotic bone repair
Source: J Nanobiotechnology. 2026 Aug 1;24:1006. doi: 10.1186/s12951-026-04879-0 (PMC13721547; doi:10.1186/s12951-026-04879-0)
Supplement: Supplementary file 1 [file 12951_2026_4879_MOESM1_ESM.docx]

**Supporting Information**

**Fig. S1.** Time-dependent dissolved oxygen (O_2_) generation profiles. Dissolved O_2_ concentrations in 200 μM H_2_O_2_ solution following incubation with LDHs or MnO_2_@LDHs for up to 120 min.

**Fig. S2.** Optimization of the MnO_2_@LDHs loading concentration in the GelMA hydrogel. **(A)** CCK-8 assay evaluating the viability of BMSCs cultured on composite hydrogels containing different concentrations of MnO_2_@LDHs (n = 4). **(B)** Cytoskeletal morphology and cell spreading of BMSCs visualized by FITC-phalloidin staining. **(C)** qPCR analysis of osteogenic marker gene expression in BMSCs (n = 4).

**Fig. S3.** Mechanical stability of the hydrogels during hydrolytic degradation. Dynamic changes in the compressive Young's modulus of the GelMA, LDHs/GelMA, and MnO_2_@LDHs/GelMA hydrogels after immersion in PBS for 0, 1, 7, and 14 days (n = 3).

**Fig. S4.** Release kinetics of bioactive ions from the composite hydrogel. Cumulative release profiles of Mg^2+^ and Mn^2+^ ions from the MnO_2_@LDHs/GelMA nanocomposite hydrogel during incubation in PBS at predetermined time points (days 1, 3, 5, 7, and 14) (n = 3).

**Fig. S5.** Cytocompatibility of the composite hydrogels. CCK-8 assays evaluating the viability of **(A)** BMSCs, **(B)** BMDMs, and **(C)** HUVECs cultured on different hydrogels for 1, 2, and 3 days (n = 4).

**Fig. S6.** Osteoinductive evaluation of hydrogel leachates. Quantitative analysis of alkaline phosphatase (ALP) activity in BMSCs cultured with conditioned leachates prepared from different hydrogels for 4 days (n = 3).

**Fig. S7.** Validation of the OP rat model and systemic biosafety evaluation *in vivo*. **(A)** 3D micro-CT reconstructions and **(B)** quantitative morphometric analysis of distal femurs in Sham and OVX rats (n=4). **(C)** H&E staining images of distal femurs in Sham and OVX rats. **(D)** ELISA quantification of serum E2 levels (n=4). **(E)** H&E staining images of major vital organs at 8 weeks post-implantation. **(F)** Routine blood parameters and **(G)** serum biochemical markers evaluation at 8 weeks post-implantation (n=4).

**Table S1.** Sequences of primers used for qPCR detection.

| Gene | | Forward sequence (5’-3’) | Reverse sequence (5’-3’) |
| --- | --- | --- | --- |
| Rat | *Alp* | AACGTGGCCAAGAACATCATCA | TGTCCATCTCCAGCCGTGTC |
|  | *Runx2* | CCATAACGGTCTTCACAAATCCT | TCTGTCTGTGCCTTCTTGGTTC |
|  | *Opn* | TTGGCTTTGCAGTCTCCTGCGG | AGGCAAGGCCGAACAGGCAAA |
|  | *Ocn* | GGTGCAGACCTAGCAGACACCA | AGGTAGCGCCGGAGTCTATTCA |
|  | *Nfatc1* | CTTCCAAGTTTCCACTCGGC | ACGCATAACTGTAGTGTTCTTCC |
|  | *Mmp9* | GCTTCTGTCCTACTCGAGCC | ACAGGCTGTACCCTTGGTCT |
|  | *Ctsk* | CTGTGGAAGAAGACCCACGG | ACTTCTTCGCTGGTCATGTCT |
|  | *Acp5* | CTCTTCTACTGAGAGGTGCGAG | ACCATCCACGTATCCATCACC |
|  | *Il-1β* | CAGCTTTCGACAGTGAGGAGA | TTGTCGAGATGCTGCTGTGA |
|  | *Il-6* | AGAGACTTCCAGCCAGTTGC | TGCCATTGCACAACTCTTTTC |
|  | *iNos* | GAAGGTCGCCAGTCGCGT | TTGCAAGAGATATCCGAGGTGG |
|  | *Tnf-α* | GATCGGTCCCAACAAGGAGG | CTTGGTGGTTTGCTACGACG |
|  | *Arg1* | TGTGCCCTCTGTCTTTTAGG | CTCCTCGAGGCTGTCCCT |
|  | *Il-10* | CCTCTGGATACAGCTGCGAC | TAGACACCTTTGTCTTGGAGCTTA |
|  | *Cd206* | CGCTGTTCAACTCTTGGACTC | CTGAACGGAGATGGCGCTTA |
|  | *Tgf-β* | CTGGAAAGGGCTCAACACCT | CTTCTCTGTGGAGCTGAAGCA |
|  | *Sirt1* | CCCAGATCCTCAAGCCATGTT | TGCTTCAATGCTGTTTCTTCTTT |
|  | *Sod2* | GCAAGGAACCACAGGCCTTA | TACAAAACACCCACCACGGG |
|  | *Cat* | GCTCCGCAATCCTACACCAT | GGACATCGGGTTTCTGAGGG |
|  | *Gapdh* | AGTGCCAGCCTCGTCTCATA | TGAACTTGCCGTGGGTAGAG |
| Human | *SIRT1* | GCTCGCCTTGCTGTGGACTTC | GTGACACAGAGATGGCTGGAACTG |
|  | *SOD2* | GCTGGAGGCTATCAAGCGTGAC | TTAGAGCAGGCGGCAATCTGTAAG |
|  | *CAT* | GTGCGGAGATTCAACACTGCCA | CGGCAATGTTCTCACACAGACG |
|  | *GAPDH* | GGAGCGAGATCCCTCCAAAAT | GGCTGTTGTCATACTTCTCATGG |

**Table S2.** Primary antibody name, source manufacturer and catalog number.

| **Primary antibody** | **Manufacture and catalog number** |
| --- | --- |
| iNOS | Proteintech, 18985-1-AP |
| CD86 | MCE, HY-P80609 |
| CD206 | Proteintech, 18704-1-AP |
| OCN | Epizyme, P106951 |
| CD31 | Servicebio, GB11063-2 |
